# The first century of *JCI* and beyond

**DOI:** 10.1172/JCI186113

**Published:** 2024-10-01

**Authors:** Elizabeth M. McNally

This issue marks the 100th anniversary of the *Journal of Clinical Investigation* (*JCI*). First published in October 1924, the *JCI* is the flagship journal of the American Society for Clinical Investigation (ASCI). The ASCI was formed in 1909, as a medical society of physician-scientists dedicated to better understanding mechanisms of human disease or what was defined as “The Science of Clinical Medicine” (1). Issues from the early years of the *JCI* described fundamental physiological mechanisms relying on observations made in humans or relevant animal species. Physiological discoveries were viewed as essential to gaining insight to active biomedical processes, and the emphasis on physiology in the early 1900s stood in contrast to the descriptions gleaned from autopsies, which were viewed as “last century” and even “the science of the dead.” As a society, the ASCI embraced a membership of investigators who were themselves active as physicians and scientists, emphasizing the role of experimentation and relying on new methods and modern techniques. Concomitant with this membership, the goal of the JCI was aimed as not only describing laboratory findings, but emphasizing papers on control and cure of disease.

One hundred years later, in 2024, the *JCI* retains this scope by publishing studies that uncover mechanisms of disease, identify potential therapeutic targets, or demonstrate the utility of disease targets in changing disease outcomes. When the *JCI* was established a century ago, the Journal’s founders felt there was not a home for this type of investigation and that publishing in the existing journals was too slow, limiting the dissemination of critical scientific information. On its 100th birthday, the *JCI* retains its core mission but through a modern lens, especially with respect to human investigation.

To recognize the many achievements of modern experimental medicine and emphasize the role that the *JCI* has played in these advancements, throughout this year we have been publishing a series of 100th anniversary Viewpoints, highlighting key discoveries from the past century. The Viewpoints cover topics mostly from the last 50 years, because it is these discoveries that have especially transformed human health and extended human lifespan. In selecting topics for these Viewpoints, the Editorial Board was drawn to many of the *JCI*’s most highly cited papers. These papers and others are notable for laying essential molecular foundations defining the science of clinical medicine and for the impact of the discoveries on human health.

The Viewpoint by Brown and Goldstein spotlights the *JCI*’s highest cited paper from 1955, which details the fractionation of lipoproteins in human serum and the application of this method to define LDL and HDL, opening up the field of human lipid biology (2). On this topic, I want to highlight a paper I find particularly intriguing that was not cited in their Viewpoint (mostly because we limit the references in these Viewpoints). In 1973, Dr. Goldstein described the inheritance patterns of serum lipid profiles in probands who survived myocardial infarction and their family members (3). As critical as the development of an assay is to the field, so is the ability to link clinical findings with laboratory tests. In this case, the authors evaluated over 2,500 participants, and from these data, they constructed pedigrees that demonstrated at least 5 distinct disorders, including familial hypercholesterolemia (FH), familial hypertriglyceridemia, and familial combined hyperlipidemia. The authors concluded that approximately 1 of every 160 individuals may carry one of these genes, underscoring the contribution to public health of these genetic findings. This paper brings together clinical medicine, laboratory science, and genetics, before the advent of modern human genetic methods. It was fibroblasts from patients with FH that enabled the subsequent identification of HMG-CoA reductase with the role of the LDL receptor and the eventual discovery of the inhibitors that would become statins. The more recent demonstration of PCSK9 inhibitors also derived from similar human genetic data, noting naturally occurring forms of PSCK9 loss-of-function mutations connected to intrinsically lower cholesterol (4).

Another Viewpoint outlines the discovery of the incretin pathway, especially glucagon-like peptide-1 (GLP-1) (5). The drugs that act on these pathways are currently transforming medicine, similar to the impact of statins on lipid management. The Viewpoint by Daniel Drucker recounts the critical papers in *JCI* over the 30 years that helped define the sites and modes of action of the GLPs for treating diabetes and control of gastric emptying and food intake (5). While initially approved to treat diabetes, GLP-1 receptor agonists impact appetite and gastric emptying as well has having effects on vascular health and inflammation. Perhaps even more interesting is their impact on obesity, which has gained the attention of the public, government, and industry for its potential reach to many aspects of health and lifestyle.

Papers in the *JCI* have had remarkable impact in nearly all branches of medicine, from neurodegeneration to novel cancer treatments to cardiovascular and renal health and infectious diseases. For example, in 1986, the elevation of natriuretic peptides in patients with heart failure was reported in the *JCI* (6), and today these tests are now routinely used to risk stratify and manage patients from the emergency room to the outpatient setting. More than 20 years ago, a *JCI* paper described the role of hypoxic mucous plaques in promoting *Pseudomonas aeruginosa* infection in cystic fibrosis (CF) (7), and it was observations like these that began to extend the lifespan of patients with CF. The *JCI* reported the importance of triple-negative breast cancer subtypes and specifically the role of targeted therapies (8), providing early rationale for precision therapies in cancer management and for this very high-risk group of patients with breast cancer. The *JCI* also published landmark papers on the importance of p53 in cancer and chemotherapy response, the combined effect of checkpoint blockade with radiotherapy, and improving CAR T cell–based treatment for lymphoma (9–11), with each of these contributions influencing clinical management and outcomes. In recent years, the *JCI* published an early report on the COVID-19 features associated with disease severity that helped guide patient management through the pandemic (12). The *JCI* also described the role of maternal fetal antibodies after vaccination with mRNA vaccines for SARS-CoV-2 (13).

## JCI for the next century

The science of medicine is increasingly mechanistic, promoted by the ability to more deeply define human disease etiologies using genetic and epigenetic data, adding in other profiling capacities, like metabolomics, lipidomics, and glycomics — letting investigators and clinicians better subclassify human disorders across all specialties of medicine. The postgenome era of the last two decades has been accompanied by massive human DNA sequencing efforts, uncovering a level of genetic heterogeneity that was unanticipated. The majority of recent FDA drug approvals have genetic underpinnings (14), and this trend is not slowing. With more ancestrally diverse datasets, we gain new knowledge that directly informs disease risk, and at the same, these data yield new opportunities. The participants who donate their samples and time to these large data acquisition efforts are themselves promoting science and, from this, society benefits.

The combined ability to access and study human materials is accompanied by better ways of imaging humans, providing greater resolution and in vivo human evidence of specific pathological pathways and their targets. There are now less invasive approaches that allow for repeated tissue and cell sampling, and serial imaging, allowing analyses of disease trajectory. Fueled by electronic medical record information and new monitoring devices (often as simple as a smart watch), we can better link molecular data to disease risk and tempo. Trajectory analyses uncover faster and slower progressors, and this ability to harness real-time, real-world data will accelerate clinical trials and improve outcomes, reducing the time to decisions regarding drug approvals and even aiding in postregulatory approval surveillance. Methods to analyze these exponentially growing data repositories are expanding. Machine learning and artificial intelligence are propelling these analyses, gleaning patterns invisible to the investigator’s eye. While there are concerns about artificial intelligence in medicine, most acknowledge that, with thoughtful application, the net benefit should make medicine safer and more efficient.

In the near term, we can expect to see advances in modeling human disease. The recent years have brought more precise and accurate 3D protein modeling (15), and cryo-electron microscopy is deciphering protein interaction information for macromolecular complexes. Cellular and tissue engineering will also inform disease outcomes and pathogenesis. Three key transformative discoveries from the early part of this century are enabling human biomedical discovery: (a) massively parallel, reduced-cost nucleic acid sequencing; (b) the capacity to generate individualized induced pluripotent stem cells from human cells; and (c) gene editing. The combination of these skills and tools makes it feasible to have engineered human organoids and tissues that better represent human conditions. These platforms will permit testing for genetic and therapeutic interventions as well as providing new disease understanding.

In 1996, the primary research content of the *JCI* became freely accessible online, making the *JCI* an original open access journal. *JCI Insight* started in 2016, and, as the much younger sister journal to the *JCI*, it has continued to grow in scope, and it also is open access. *JCI Insight*, like the *JCI*, is a journal run by active scientists to serve the needs of the scientific community. We welcome our colleagues at the University of Pittsburgh who are stewards for *JCI Insight* over the next 5 years under the guidance of Dr. Oliver Eickelberg. We also commend Dr. Kathleen Collins and her superb team at the University of Michigan for their work leading *JCI Insight* over the last five years. We look forward to the next 100 years and reporting the scientific discoveries that will change human health.

## Version 1 10/01/2024

Electronic publication
